# Interprofessional Collaboration for Implementing Evidence-Based Practices n Nursing Homes

**DOI:** 10.1093/geroni/igaf122.313

**Published:** 2025-12-31

**Authors:** Katherine Abbott, Natalie Douglas

## Abstract

Effective long-term care (LTC) for older adults requires innovative approaches to interprofessional collaboration, team coaching, and the integration of evidence-based interventions. This symposium presents five studies exploring strategies to enhance LTC by improving communication, implementation processes, and care delivery. The first study examines care professionals’ perspectives on electronic health records (EHRs) as a tool for interprofessional collaboration in Dutch nursing homes, identifying key areas for optimization. The second study explores the role of team coaching in implementing psychosocial interventions to improve sleep, mobility, and fall prevention in nursing home residents. Through qualitative analyses, it highlights facilitators and barriers to team coaching adoption. The third study investigates the characteristics and mechanisms of action of clinical site champions in a Veterans Affairs project, revealing critical traits that contribute to successful implementation of goals-of-care conversations. The fourth study details the engagement of a Community Advisory Board (CAB) in refining a hospital-to-skilled nursing facility transition intervention, emphasizing the importance of family caregiver involvement. Finally, the fifth study evaluates the impact of the Individualized Positive Psychosocial Interaction program on nursing home staff care partners, demonstrating improvements in knowledge, self-efficacy, well-being, and meaningfulness of care interactions. The Discussant, Dr. Natalie Douglas, will discuss the implications of these projects for researchers, clinicians, and policymakers seeking to enhance the quality of care for older adults in LTC settings. Together, these studies highlight the importance of interdisciplinary collaboration, implementation strategies, and person-centered interventions in improving LTC outcomes.

